# Effects of Aerobic Exercise on Blood Pressure Response during Exercise

**Published:** 2019-09

**Authors:** Il-Kon KIM, So-Hyung KANG

**Affiliations:** Sports and Health Care Major, Mokwon University, Daejeon Metropolitan City, Republic of Korea

## Dear Editor-in-Chief

Hypertension is one of the most important factors considered in cardiovascular diseases because of its high prevalence, medical cost, etc. (1). The most effective method to prevent hypertension is exercising everyday (2). Blood pressure frequently varies because of various reasons, such as ambient temperature, environment, and stress level, and especially changes dramatically during exercise. Blood pressure response during and after exercise can predict the risk associated with coronary heart disease (CHD). In healthy middle-aged males, long-term CHD risk is increased if the systolic blood pressure is >180 mmHg when exercising with an appropriate load (3).

Thus, it is assumed to be more effective if it were possible to people should become more aware of the necessity of exercise by measuring blood pressure response during exercise as changes in blood pressure are obvious during exercise. This study, which classified middle-aged Korean males into two groups, those with >5 years of exercise experience and those with no experience, aimed to compare and analyze blood pressure response during and after exercise and investigate the effect of ordinary exercise practice on blood pressure response.

We enrolled 26 middle-aged Korean males with no history of drugs, smoking, or drinking, and they were divided into the exercise group that included participants who regularly performed aerobic and anaerobic exercises for over 5 years and the non-exercise group wherein participants had not exercised in 5 years (13 males with entirely no exercise).

The study was approved by the Mokwon University. Furthermore, the subjects were informed sufficiently regarding the research.

The participants’ characteristics are presented in Table 1.

**Table 1:** Characteristics of participants

***Variables***	***Non-exercise group (n=13)***	***Exercise group (n=13)***
Age (yr)	47.76±4.93	48.15±5.71
Height (cm)	170.61±6.07	173.38±3.92
Weight (kg)	74.07±11.45	72.23±10.72
Body fat (%)	27.07±3.63	24.53±2.53
Heart rate (beats/min)	71.84±6.21	73.38±4.82
Systolic blood pressure (mmHg)	121.69±4.87	122.53±5.47
Diastolic blood pressure (mmHg)	81.69±3.14	80.07±3.47

Data are presented as mean ± standard deviation

An expert prescribed the exercises and helped in the research during the experiment period. For more accurate measurement of resting blood pressure and heart rate, blood pressure was measured after 5∼10 minutes of participants resting on chairs. A well-trained nurse measured blood pressure and heart rate on the right brachial artery by using a sphygmomanometer (HEM-7320, Omron, Kyoto, Japan). Maximal graded exercise test was conducted at a community healthcare center in Daejeon metropolitan city to accurately measure exercise intensity of each individual (4). The blood pressure was measured twice, 30 minutes before exercise and 20 minutes after completing the exercise by placing the cuff of the automated blood pressure device (HEM-7320, Omron, Kyoto, Japan) on the right brachial artery for relative measurement during and after the exercise. The single design exercise method employed was aerobic exercise with 4-METs of exercise intensity that consisted of 5 minutes of warm-up, 50 minutes of main exercise, and 5 minutes of cool-down by using a treadmill (DEJ4EL, Jog Forma, Italy) according to the exercise capacity of each individual based on maximum graded exercise test according to American College of Sports Medicine’s Guidelines (5).

The results of this study were expressed as mean ± standard deviation by using SPSS ver. 21.0 (IBM Corp., Armonk, NY, USA). Repeated-measures analysis of variance was conducted to compare and analyze the systolic and diastolic blood pressure measured 30 minutes after start of exercise and 20 minutes after completion of exercise of 2 groups who were divided by exercise experience. Statistical significance was set at *P*<0.05.

Table 2 shows statistically significant result with respect to change in systolic blood pressure between the non-exercise and exercise groups (*P*=0.011), but there was no significance in diastolic blood pressure (*P*=0.105).

**Table 2:** Change in blood pressure during exercise according to exercise practice status

***Groups***	***Systolic Blood Pressure (mmHg)***				***Diastolic Blood Pressure (mmHg)***			
	***1st***	***2nd***	***Interaction***		***1st***	***2nd***	***Interaction***	
			***F***	***p***			***F***	***p***
NEG (n=13)	200.31±8.08	159.46±8.60	7.578	0.011*	104.6±11.01	96.62±3.77	2.842	0.105
EG (n=13)	157.62±8.13	123.69±4.34			83.85±4.10	78.62±3.92		

Data are presented as mean ± standard deviation

NEG: non-exercise group, EG: exercise group

1st: 30 minutes after start of exercise; 2nd: 20 minutes after completion of exercise

* *P*<0.05; tested by repeated-measures analyses of variance

In conclusion, the increase in systolic and diastolic blood pressure level is low in the exercise group than in the non-exercise group as well as better recovery of systolic and diastolic blood pressure after exercise. However, statistical significance was shown in only systolic blood pressure result between the exercise and non-exercise groups. From these results, we believe that exercise affects increase in blood pressure during exercise especially that of systolic blood pressure and it is assumed that exercise has a positive effect on the elasticity of blood vessels. Additionally, exercise has an obvious effect in reducing the risk of accidents from vascular disease during exercise as there is no dramatic change in blood pressure.
